# A Novel Controlled PTEN-Knockout Mouse Model for Prostate Cancer Study

**DOI:** 10.3389/fmolb.2021.696537

**Published:** 2021-06-03

**Authors:** Sen Liu, Bing Zhang, Brian G. Rowan, S. Michal Jazwinski, Asim B. Abdel-Mageed, Chad Steele, Alun R. Wang, Oliver Sartor, Tianhua Niu, Qiuyang Zhang

**Affiliations:** ^1^ Department of Structural and Cellular Biology, Tulane University School of Medicine, New Orleans, LA, United States; ^2^ Medical Laboratory of ShenZhen LuoHu People’s Hospital, Shenzhen, China; ^3^ Department of Medicine, Tulane University School of Medicine, New Orleans, LA, United States; ^4^ Tulane Center for Aging, Tulane University School of Medicine, New Orleans, LA, United States; ^5^ Department of Urology, Tulane University School of Medicine, New Orleans, LA, United States; ^6^ Department of Microbiology and Immunology, Tulane University School of Medicine, New Orleans, LA, United States; ^7^ Department of Pathology and Laboratory Medicine, Tulane University School of Medicine, New Orleans, LA, United States; ^8^ Tulane Cancer Center, Tulane University School of Medicine, New Orleans, LA, United States; ^9^ Department of Biochemistry and Molecular Biology, Tulane University School of Medicine, New Orleans, LA, United States

**Keywords:** cre-expressing adenovirus, age, prostate cancer, Pten, mouse models

## Abstract

Prostate cancer (PCa) is associated with advanced age, but how age contributes to prostate carcinogenesis remains unknown. The prostate-specific Pten conditional knockout mouse model closely imitates human PCa initiation and progression. To better understand how age impacts PCa in an experimental model, we have generated a spatially and temporally controlled Pten-null PCa murine model at different ages (aged vs. non-aged) of adult mice. Here, we present a protocol to inject the Cre-expressing adenovirus with luciferin tag, intraductally, into the prostate anterior lobes of Pten-floxed mice; Pten-loss will be triggered post-Cre expression at different ages. *In vivo* imaging of luciferin signal following viral infection confirmed successful delivery of the virus and Cre activity. Immunohistochemical staining confirmed prostate epithelial-specific expression of Cre recombinase and the loss of Pten and activation of P-Akt, P-S6, and P-4E-BP1. The Cre-expression, Pten ablation, and activated PI3K/AKT/mTOR pathways were limited to the prostate epithelium. All mice developed prostatic epithelial hyperplasia within 4 weeks after Pten ablation and prostatic intraepithelial neoplasia (PIN) within 8 weeks post-Pten ablation. Some PINs had progressed to invasive adenocarcinoma at 8–16 weeks post-Pten ablation. Aged mice exhibited significantly accelerated PI3K/AKT/mTOR signaling and increased PCa onset and progression compared to young mice. The viral infection success rate is ∼80%. This model will be beneficial for investigations of cancer-related to aging.

## Introduction

Prostate cancer (PCa) is the second most commonly diagnosed noncutaneous cancer and the fifth leading cause of cancer death in men worldwide (Bray et al., 2018). Among men in the United States, it is the most common cancer, accounting for 26% of cancer diagnoses (Siegel et al., 2021), and the second leading cause of cancer deaths accounting for about 34,000 deaths annually (Siegel et al., 2021). PCa is particularly significant in older men given the high incidence and prevalence of disease and mortality in this group of patients (Stangelberger et al., 2008). PCa is a hormone-sensitive disease. Continued exposure to environmental and dietary factors may also lead to the accumulation of genetic and epigenetic changes during the aging process, resulting in tumor promoter and tumor suppressor genes altered expression and/or activity (Vaidyanathan et al., 2016). However, the increased incidence and mortality of PCa in older men is only partially known. Therefore, understanding how aging influences disease risk is highly important.

Genetically engineered mouse models (GEMMs) have been valuable tools for defining the cellular and molecular mechanisms responsible for PCa initiation and progression in the context of the whole organism and in the native milieu in which tumors arise, which might otherwise be difficult to characterize using other types of animal models (Grabowska et al., 2014; Rea et al., 2016; Arriaga and Abate-Shen, 2019). However, unlike the murine models of PCa, human PCa is heterogeneous and does not typically develop from germline genetic changes (Bostwick et al., 1998). Although several temporally controlled murine models were recently developed in adult mice (Luchman et al., 2008a; Ratnacaram et al., 2008; Luchman et al., 2008b), the models still use the very young mice (before two months of age). Using a PCa animal model generated in adult mice to understand PCa initiation and progression would be more applicable to human PCa initiation and progression, as PCa is associated with advanced age. Most studies of human PCa in laboratory animals are done using young animals; tumorigenesis in most GEMMs is too rapid to permit aging-related comparisons. Therefore, it is important to develop an age-dependent PCa mouse model to understand the contribution of age to PCa better.

The phosphatase and tensin homolog deleted on chromosome 10 (PTEN) is a dual-specificity protein and lipid phosphatase that, in humans, is encoded by the tumor suppressor gene PTEN. Mutations in this gene are a step in the development of many human tumors (Song et al., 2012). Extensive studies by numerous groups have demonstrated that PTEN regulates cell growth, apoptosis, and proliferation. It supports cell metabolism, polarity, motility, cancer “stem-ness,” and stromal-epithelial interactions (Song et al., 2012). Monoallelic loss of PTEN is present in up to 60% of localized PCa, and complete loss of PTEN in PCa is linked to metastasis and androgen-independent progression (Cairns et al., 1997; Suzuki et al., 1998; Wang et al., 1998). Therefore, the prostate-specific Pten conditional knockout (cKO) mouse model (Wang et al., 2003), which closely mimics the formation and growth of human PCa, has become an established PCa preclinical model. This model and its application have provided vital information on human PCa progression (Zhang et al., 2012; Zhang et al., 2014; Liu et al., 2016; Qu et al., 2016; Zhang et al., 2017a; Zhang et al., 2017b). However, as Pten deletion in this model is triggered in the 2-week-old prostate, it is difficult to distinguish the extent to which the onset and progression of PCa is due to acceleration by the normal aging process or manifestation of PCa pathologies over time (Zhang et al., 2020). We have previously aged these mice to ∼70 weeks-old (aged) and observed that they exhibit severe onset and progression of PCa compared to young mice. Still, the manifestation of PCa pathology can only be attributed to the passage of time and not necessarily the effects of aging.

Here, we created a novel spatially and temporally controlled *Pten* KO (referred to as *Pten* ^*adcre+*^) mouse model using a virus-assisted *in vivo* conditional KO approach. The prostate-specific Cre-LoxP gene switching was generated *via* intraductal delivery of adenovirus to the anterior prostate lobes (Maddison et al., 2000; Leow et al., 2005). Adenovirus is a DNA virus that does not integrate into the host genome. It infects dividing and nondividing cells, leading to transient high-level protein expression (Tao et al., 2014). Using the intraductal delivery method, we obtained prostate epithelial-specific infection of Cre-expressing adenovirus, leading to the deletion of the floxed Pten gene in the prostate epithelium. By comparing the onset and progression of PCa generated at different ages using this method, we confirmed that aged mice develop a higher incidence and progression of PCa compared to young mice. This study aims to provide a novel approach for developing age-related murine models for PCa, emphasizing the effect of aging on prostate carcinogenesis.

## Materials and Methods

### Mice

*Pten* ^*loxp/loxp*^ (*Pten* ^*L/L*^) mice (strain name: C; 129S4-*Pten* ^*tm1Hwu*^/J; genetic background: 129S4/SvJae^*^BALB/c) were obtained from the Jackson Laboratory (Bar Harbor, ME) and were housed under specific pathogen-free conditions at the animal facility at Tulane University. *Pten* ^*L/L*^ mice were kept in different age batches, aged 12–28, 44–60, and 96–112 weeks, representing young, middle-aged, and aged groups, respectively (Liu et al., 2020). Scaling of the mouse to human age was accomplished using published criteria (Carnes et al., 2003). The animal study was approved by the Institutional Animal Care and Use Committee of Tulane University.

### *In vivo* Delivery of Adeno-Cre-Luc Virus in Pten^L/L^ Mice

**FIGURE 1:**
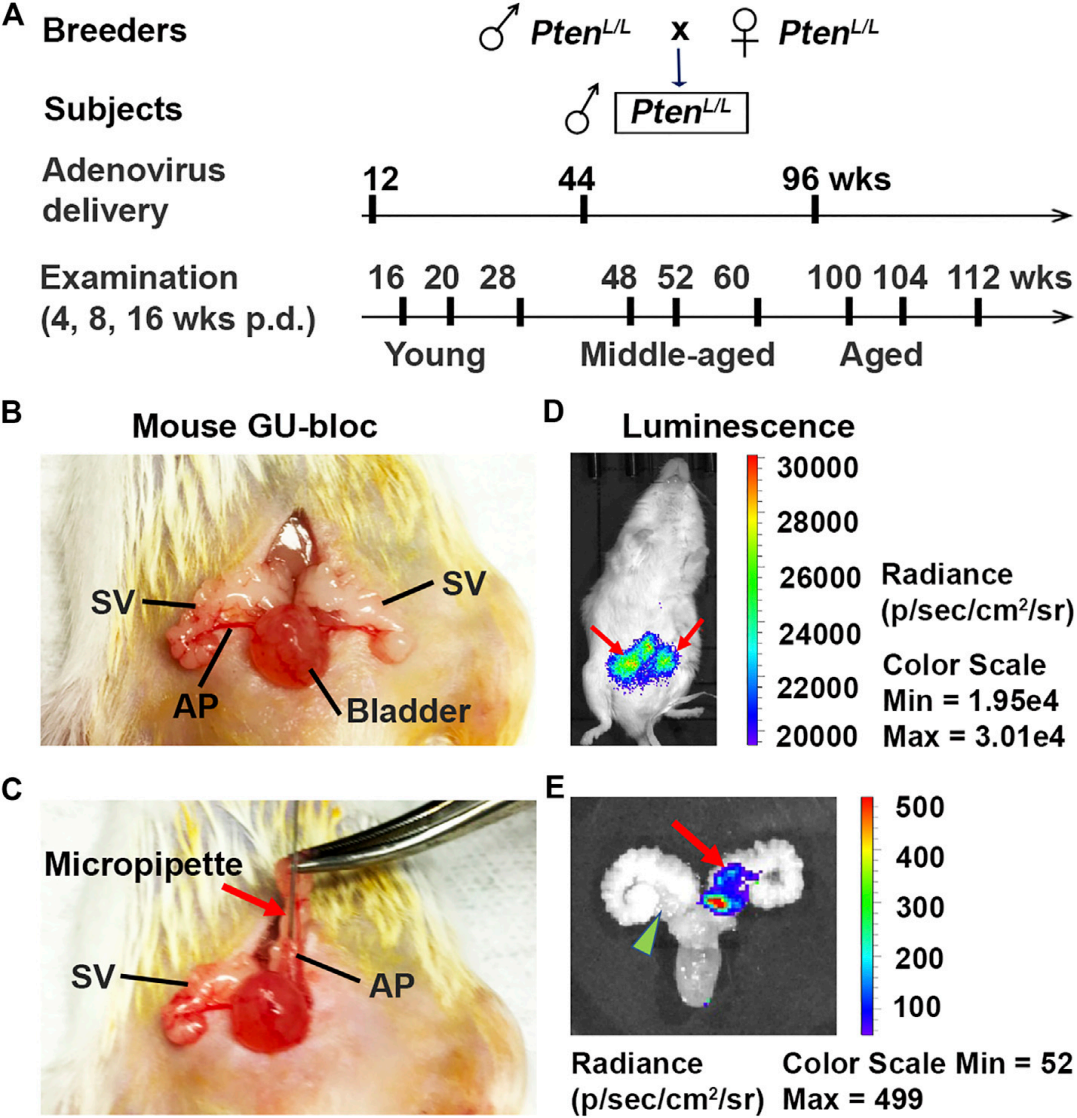
Establishment of Pten knockout (*Pten^adcre+^*) mouse model. **(A)** The mouse breeding strategy, adenovirus dosing schedule, and ages (young, middle-aged, and aged) at which mice were examined; p.d., post-delivery. **(B–C)** The approach of intraductal delivery of adenovirus. The seminal vesicle (SV) was briefly exteriorized, and a micropipette was carefully guided into a primary duct in the prostate anterior lobe (AP); arrow indicates micropipette. **(D)** Live image of luciferin signals 5 days p.d.; arrows indicate luciferin signals. **(E)** Image of luciferin signal in the APs 5 days p.d.; arrow indicates luciferin signal in an AP; arrowhead indicates non-injected AP.

The *Pten* ^*L/L*^ young (12 weeks old), middle-aged (44 weeks old), and aged (96 weeks old) male mice were deeply anesthetized with 2–4% isoflurane in oxygen. All surgical procedures were performed under sterile conditions and per the guidelines of the Institutional Animal Care and Use Committee at Tulane University. The furred mice, prepared by a surgeon wearing a mask and sterile gloves, were shaved prior to a skin application of a betadine solution and 70% ethanol. An analgesic drug (Buprenorphine) was administered to the mice before incision. Then a small incision (1.5 cm) was made to expose the seminal vesicles (SVs). The SVs were exteriorized only briefly for injection to prevent dehydration of the tissue. Since adenovirus was used, we conducted all injections within a biosafety hood with a modified sash (Leow et al., 2005). A LEICA S9D Stereo Zoom microscope was used to facilitate surgical maneuvers. The anesthetized mice were placed on a heating pad during surgery, and 5–10 μl (4∼8 x 10^^6^ PFU/g of body weight) of viral solution (1 x 10^^10^ PFU/ml) were intraductally delivered into the anterior prostate (AP) using a micropipette (Hamilton Company, Reno, NV). The Cre-expressing Adenovirus (Cat. No. 1705, named as Ad-Luc-Cre) was obtained from Vector Biolabs, Malvern, PA. After injection, the peritoneum was sutured using a 5-0 vicryl suture, and the skin was closed using a 5-0 nylon suture (Leow et al., 2005; Figures 1A–C).

### Live Imaging of Mice and Virus Infection Rate Calculation

Five days post-surgery, the mice were subjected to live imaging using the IVIS-Lumina XRMS *in vivo* imaging system (PerkinElmer, Inc., Waltham, MA). Animals were anesthetized with 2–4% isoflurane in oxygen. D-luciferin (15 mg/ml) (Gold Biotechnology, St. Louis, MO) was injected intraperitoneally at a dose of 10 μl/g of body weight. Image acquisition was started immediately with a series of images within 30 min. The peak light emission intensity represents the injected Adeno-Luc-Cre (Figures 1D,E). The mouse APs that have detectable luciferin signals were counted. The success infection rate was calculated as the number of luciferin signals divided by the total number of injected APs.

### Quantitative Reverse Transcription-PCR (qRT-PCR)

qRT-PCR was performed in the mouse anterior prostate tissues from young and middle-aged mice to detect the Pten gene knockout efficiency as described (Tian et al., 2019; Liu et al., 2020). Total RNA was isolated according to the instructions of an RNeasy Mini Kit (Qiagen) with on-membrane DNase I digestion to avoid genomic DNA contamination (Chen et al., 2015). cDNA was made from total RNA using an iScript cDNA synthesis kit (Bio-Rad Laboratories). The primer sequences were obtained from Eurofins. Mouse glyceraldehyde-3-phosphate dehydrogenase (*Gapdh)* forward: 5′-TGC​ACC​ACC​AAC​TGC​TTA​G-3′, reverse: 5′-GGA​TGC​AGG​GAT​GAT​GTT​C-3′; Pten forward: 5′- TGA​AGA​CCA​TAA​CCC​ACC​ACA-3′, reverse: 5′-TCA​TAC​ACC​AGT​CCG​TCC​CT. Results were normalized to *Gapdh* levels using the formula ΔCt (Cycle threshold) = Ct of target gene - Ct of *Gapdh*. The mRNA level of the control group (mice injected Ad-CMV-Luc) was used as the baseline; therefore, ∆∆Ct was calculated using the formula ∆∆Ct = ∆Ct of target gene - ∆Ct of the baseline. The fold change of mRNA level was calculated as fold = 2^−∆∆Ct^ (Chen et al., 2015).

### Histopathologic Assessment and Inflammation Examination

Mice were weighed and euthanized at the designated time points. The genitourinary blocs (GU-blocs), which consist of the prostate lobes, seminal vesicles, ampullary glands, bladder, proximal ductus deferens, and proximal urethra, were excised *en bloc* (Shappell et al., 2004; Chan et al., 2007), carefully dissected, photographed, weighed with an empty bladder, and fixed, as described previously (Shappell et al., 2004; Zhang et al., 2012). Twenty-eight 4 μm consecutive sections of each prostate were cut, and eight sections (from every seventh section on) per sample were stained with hematoxylin and eosin (H&E) for histopathologic assessment in a group-blinded fashion according to the Bar Harbor Classification (Shappell et al., 2004; Chan et al., 2007). The prostatic glands in each AP were assessed under low- and high-power magnification. Approximately 27–94 prostatic glands in each prostate were counted, with over 500 prostatic glands in 5–10 APs per group. The number of inflammatory cells in the stroma space between the prostatic glands was counted in six high-power fields (× 400 magnification) of each AP, and the average number of inflammatory cells per high-power field in 10 mouse APs per group was compared (Zhang et al., 2012; Zhang et al., 2014; Liu et al., 2020).

### Immunohistochemical and Terminal Deoxynucleotidyl Transferase-Mediated dUTP Nick and Labeling Staining

IHC staining was performed according to previously established protocols (Zhang et al., 2012; Zhang et al., 2014; Zhang et al., 2017a; Zhang et al., 2017b; Tian et al., 2019; Liu et al., 2020), using VECTSTAIN ABC kits and DAB Substrate Kits (Vector Laboratories, Burlingame, CA, United States). The antibodies used were rabbit anti-Cre (1:500), rabbit anti-P-S6 (1:1,000), rabbit anti-P-4E-BP1 (1:500), rabbit anti-Pten (1:200), and rabbit anti-P-Akt (1:50, Cell Signaling Technology, Danvers, MA), rabbit anti-Ki-67 (1:200, Millipore, Burlington, MA), and rabbit anti-laminin (1:200; Sigma Aldrich, St. Louis, MO). Terminal deoxynucleotidyl transferase-mediated dUTP nick end labeling (TUNEL) staining was conducted with TACS XL Blue Label *In Situ* Apoptosis Detection Kits (Trevigen, Gaithersburg, MD) according to the manufacturer's instructions and previously established protocols (Zhang et al., 2012; Zhang et al., 2014; Zhang et al., 2017a; Zhang et al., 2017b). To count the P-S6-, Ki-67-, and TUNEL-positive cells, three animals from each age group were randomly selected, and prostate sections from each animal were stained. Approximately 300 epithelial cells per field of 5**–**6 high-power fields (x200 magnification) of each AP were counted. The percentage of positive cells was calculated as the number of positive cells divided by the total number of cells (Zhang et al., 2012; Zhang et al., 2014; Zhang et al., 2017a; Zhang et al., 2017b).

### Statistics

Statistical analysis was performed using SAS Software (Cary, NC). Data were analyzed using the Student’s t-test when comparing two conditions. One-way analysis of variance (ANOVA) followed by a Tukey post-hoc comparison test was performed on comparisons of more than two conditions such as *in vivo* mice GU-bloc/BW ratio, histopathology, cellular proliferation at different ages, etcetera. All *in vitro* experiments were repeated at least three times (Liu et al., 2020). Data are presented if not indicated elsewhere as mean ± standard error of the mean (SEM). Statistical significance is indicated in all figures by the following annotations: *p < 0.05; **p < 0.01; ***p < 0.001; ****p < 0.0001; ns = not significant.

## Results

### Generation of Cre-Expressing Adenovirus-Mediated Ablation of Pten (*Pten* ^*adcre+*^) in Anterior Prostatic Epithelial Cells of Adult Pten^L/L^ Mice at Different Ages

To better understand the impact of age on prostate carcinogenesis, we created a spatially and temporally controlled Pten ablation (*Pten^adcre+^*) in the mouse prostatic epithelium *via* intraductal delivery of the Cre-expressing adenovirus directly into the APs of Pten-floxed mice at different ages (Leow et al., 2005). The animal breeding, adenovirus approach *in vivo* delivery, and live imaging of luciferin signal 5 days post-delivery (p.d.) are shown in Figure 1. Unlike in humans, the mouse prostate is not a single anatomical structure, but an organ comprised of four pairs of lobes located circumferentially around the urethra. These lobes are the anterior, dorsal, ventral, and lateral lobes. The prostate anterior lobes, also named as “coagulating glands,” are translucent and bilaterally attached to the seminal vesicles' lesser curvature, cranially to the other prostate lobes. As in humans, the mouse prostate contains ducts and glands (acini) with epithelial cell types that include luminal cells, basal cells, and neuroendocrine cells (Roy-Burman et al., 2004). Under the dissection microscope, the prostatic ducts’ translucent outlines, including primary and secondary ducts of the AP, were clearly visualized (Figures 1B,C). This feature allowed us to identify the primary duct and insert the micropipette filled with the virus directly into this duct (Leow et al., 2005). The secondary duct was filled from the primary duct. To determine intraductal injection efficiency, we analyzed the luciferin signal using the IVIS^®^ Lumina XRMS imaging system 5 days post-injection (Figures 1D,E). After practicing injections on several adult mice, we injected 5–9 mice including 10–18 APs for each age group. We achieved an 80% success rate (72 of 90 APs have luciferin signals; Figure 1E each AP was counted). No leakage into the stromal area and other adjacent organs was detected. Although the injection site (primary duct) is very close to the urethra and bladder, we did not detect significant weight loss or urine obstruction in these mice, suggesting that the surgical procedure did not result in postoperative complications (Leow et al., 2005).

### Specific Expression of Cre-recombinase and PI3K/mTOR Pathway Activation in the Prostate Epithelium Post-Ad-Cre-Luc Virus Delivery at Different Time Points

**FIGURE 2:**
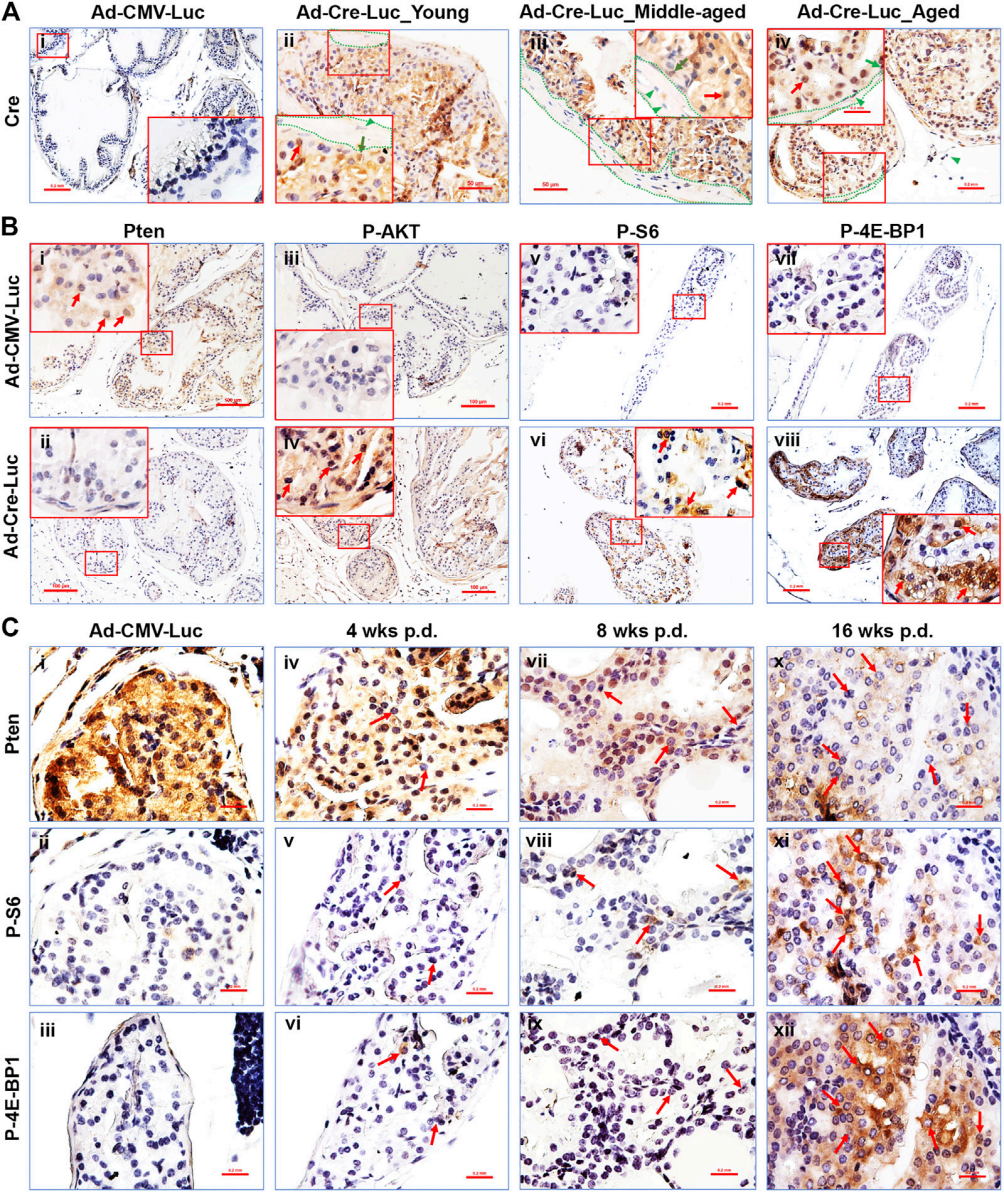
The PI3K/mTOR signaling pathways’ downstream components are activated in the AP of Pten^L/L^ mice post Cre-expressing adenovirus delivery. **(A)** Representative IHC staining of the Cre protein in control adenovirus (Ad-CMV-Luc) recipient mouse AP tissue 4 weeks p.d. **(i)**, original magnification, x100, insert, x400) and in Cre-expressing adenovirus (Ad-Cre-Luc) recipient mouse AP tissue 16 weeks p.d. **(ii–iv)**; original magnification, x200; insert, x400; red arrows, epithelial cells; green arrows, basal cells; dotted lines, stroma, green arrowheads, stroma cells. **(B)** Representative IHC staining of the Pten **(i,ii)**, P-AKT **(iii,iv)**, P-S6 **(v,vi)**, and P-4E-BP1 **(vii,viii)** proteins in control adenovirus (Ad-CMV-Luc) and Cre-expressing adenovirus (Ad-Cre-Luc) recipient mouse AP tissue 8 weeks p.d. **(i–iv)** and 16 weeks p.d. **(v–viii)**; original magnification, x100; insert, x400; arrows indicate positive cells. **(C)** Serial sections show that Pten loss in the prostatic epithelium is associated with an increase in P-S6 and P-4E-BP1 protein levels in the preneoplastic phase of *Pten* ^*adcre+*^ mice; arrows indicate cells that lost Pten and had activated P-S6 and P-4E-BP1 cells.

To determine the Ad-Cre-Luc virus infection efficiency, we performed IHC staining for Cre-recombinase (referred to as Cre hereafter) in the APs of the prostates at 4, 8, and 16 weeks post-delivery (p.d.) We observed high Cre expression levels in the prostatic epithelium in Ad-Cre-Luc virus injected mice compared to control Ad-CMV-Luc virus injected mice (Figures 2Ai–iv). The Cre-positive cells were highly localized to the prostatic epithelium with both luminal (red arrows) and basal cells (green arrows) but excluded from the stroma (dotted lines include the stroma surround prostate glands and arrowheads indicate stroma cells). These results were consistent with a previous study (Leow et al., 2005) and indicated that the adenovirus infected epithelial cells but not stromal cells.

We performed IHC staining for the Pten protein to measure Pten gene deletion in the prostate epithelium post-Ad-Cre-Luc virus delivery. Pten ablation was detected only in the prostate epithelium of mice with Ad-Cre-Luc virus delivery (Figure 2Bii). The PI3K/mTOR pathway components are known to be affected by the loss of Pten (Hollander et al., 2011; Fruman et al., 2017; Lee et al., 2018; Papa and Pandolfi, 2019). To measure activity of the PI3K pathway, we performed IHC staining for the phospho-AKT (P-AKT), phospho-S6 (P-S6), and phospho-4E-BP1 (P-4E-BP1). Pten-deficient prostate epithelium (Figure 2Bii) exhibited increased levels of P-AKT (Figure 2Biv) (Figures 2Bii,iv are consecutive sections), P-S6 (Figure 2Bvi), and P-4E-BP1 (Figure 2Bviii), indicating that the AKT/mTOR/S6K and AKT/mTOR/4EBP1 axes were activated in the prostatic epithelium. No Pten loss (Figure 2Bi), and no P-AKT (Figure 2Biii), P-S6 (Figure 2Bv), and P-4E-BP1 (Figure 2Bvii) immunostaining was observed in the mouse prostate of control Ad-CMV-Luc delivered mice. No significant Pten loss was observed in the other lobes of the Ad-Cre-Luc virus infected prostates (data not shown).

By performing IHC staining in serial sections, we found that the prostatic epithelial cells and regions lacking Pten expressed high levels of P-AKT (Supplementary Figure S1Biv,vi,viii), P-S6 (Figures 2Cv,viii,xi), and P-4E-BP1 (Figures 2Cvi,ix,xii). This phenomenon was observed within single cells at the earliest time points, becoming more dramatic within Pten-deficient cells contained within hyperplastic and prostatic epithelial neoplasia lesion regions (Luchman et al., 2008) (arrows in Figure 2C). These results demonstrated a direct correlation between Pten-deficiency and increased P-AKT, P-S6 (Luchman et al., 2008a), and P-4E-BP1 activities even in pre-neoplastic epithelial cell lesions. We also found the expression levels of P-AKT, P-S6, and P-4E-BP1 were also increased with the increased Pten loss in pre-neoplastic epithelial cell lesions (Figures 2B,C). These proteins’ expression levels were also increased with time elapsed post virus delivery (Figures 2B,C). To further test the Ad-Cre-Luc-mediated excision efficiency of floxed Pten gene, the mRNA levels of Pten in the anterior prostate tissues of young and middle-aged mice 16 weeks p.d were detected by qRT-PCR. Our results showed an average 55–75% and 50–80% of floxed Pten gene were recombined in young and middle-aged mice respectively (Supplementary Figure S2). There was no significant difference between the middle-aged and young mice (Supplementary Figure S2).

### The Ad-Cre-Luc Mediated Ablation of Pten in Adult Mouse's Anterior Prostatic Epithelial Cells Leads to Hyperplasia That Progresses Through PIN to Adenocarcinoma

The Ad-CMV-Luc or Ad-Cre-Luc was delivered to Pten-floxed mouse AP starting at 12, 44, and 96 weeks of age, respectively, and then mice were sacrificed at either 4, 8, or 16 weeks p.d.

**FIGURE 3:**
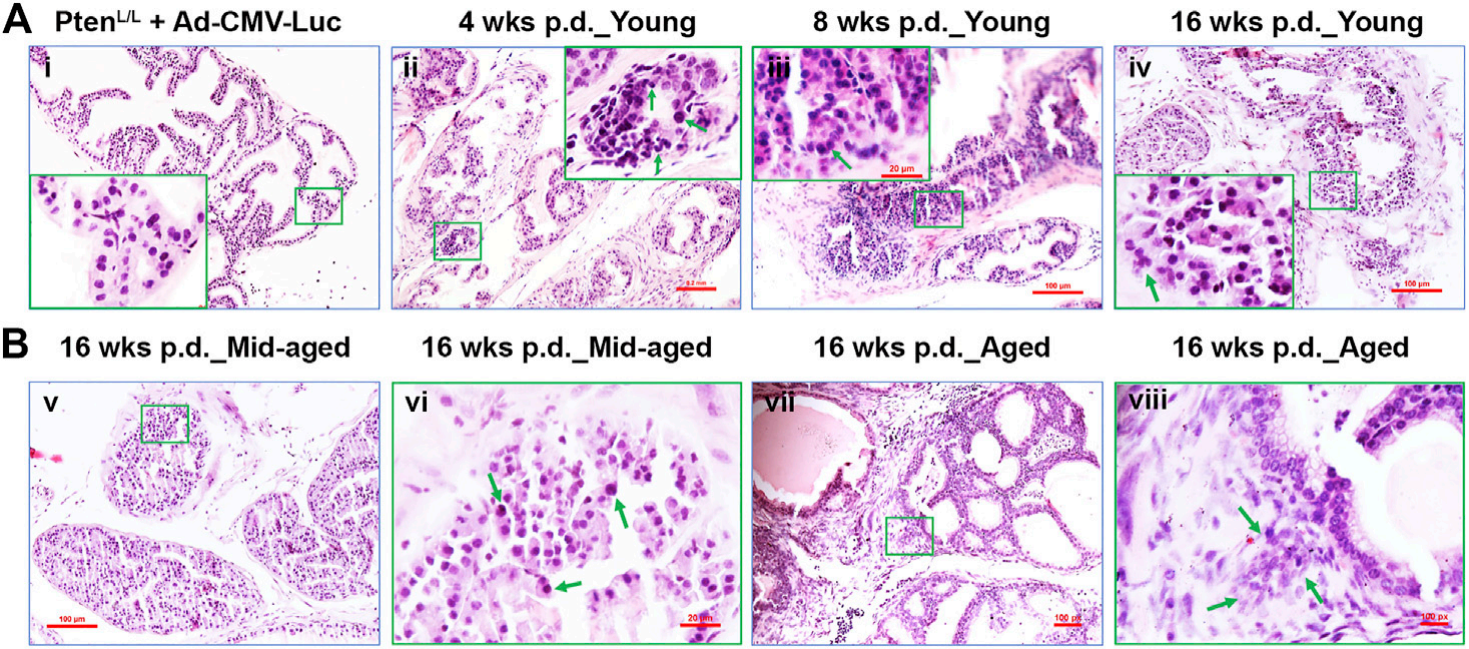
The transition from the earliest transformation stages to occur progressively in *Pten* ^*adcre+*^ mice post-Cre delivery. **(A)** Representative H&E staining of the control adenovirus (Ad-CMV-Luc) and the Cre-expessing adenovirus (Ad-Cre-Luc) recipient mouse AP tissues 4, 8, and 16 weeks p.d.; **(Ai)**, normal prostate glands were shown, middle-aged and aged mice exhibited similar results; hyperplasia **(Aii)**, prostatic intraepithelial neoplasia (PIN, **Aiii**), and microinvasive cancer **(Aiv)** were observed at 4, 8, and 16 weeks p.d.; original amplification, x100; insert, x400; arrows indicate the neoplastic cells or PIN lesion or microinvasive site. **(B)** Representative H&E staining of the Cre-expressing adenovirus (Ad-Cre-Luc) recipient mouse AP tissues 16 weeks p.d. of middle-aged and aged mice; **(Bv,vii)**, magnification, x100; **(Bvi,viii)**, magnification, x400; arrows indicate atypia cells or invasive site.

All of the Ad-CMV-Luc recipient (control) mice of the young group were similar to the age-matched wild-type mice (Figure 3Ai), thus demonstrating that the presence of adenovirus and floxed Pten did not affect prostate development and did not induce any pathological changes on their own in the adult mouse prostate (Leow et al., 2005; Ratnacaram et al., 2008). Similarly, all the middle-aged and aged group of Ad-CMV-Luc recipient mice were also similar to the age-matched wild-type mice (data not shown).

In contrast, all the *Pten* ^*adcre+*^ mice developed focal hyperplasia at 4 weeks p.d. of Ad-Cre-Luc (Table 1; Figure 3Aii, arrows indicate atypia cells). Recognizable features of PIN included proliferation of large atypical cells within pre-existing prostatic glands with enlarged nuclei, hyperchromasia, and prominent nucleoli (Shappell et al., 2004; Ratnacaram et al., 2008). In the young group, 1 of 4 APs displayed PIN lesions; of the middle-aged group, 3 of 4 APs displayed PINs; and in the aged group, 4 of 4 APs displayed PINs (Table 1). Respectively, ∼22, 45, and 55% of glands displayed hyperplasia and PINs (data not shown).

**TABLE 1:** Morphologic alterations in the APs of *Pten^adcre+^* mice at different time points post adenovirus delivery.

Mouse genotype	4 weeks p.d	8 weeks p.d	16 weeks p.d
Control^a^ mice	4 of 4 APs normal	4 of 4 APs normal	4 of 4 APs normal
*Pten^adcre+^* mice (young group)	4 of 4 APs focal hyperplasia, 1 of 4 APs PIN	8 of 9 APs hyperplasia or PIN, 2 of 18 APs micro-invasive cancer	4 of 4 APs hyperplasia or PIN, 3 of 4 APs micro-invasive cancer
*Pten^adcre+^* mice (middle-aged group)	4 of 4 APs focal hyperplasia, 3 of 4 APs PIN	7 of 8 APs hyperplasia or PIN, 4 of 8 APs micro-invasive cancer	4 of 4 APs hyperplasia or PIN, 3 of 4 APs invasive cancer
*Pten^adcre+^* mice (aged group)	4 of 4 APs focal hyperplasia, 4 of APs PIN	4 of 4 APs hyperplasia or PIN, 4 of 4 APs micro-invasive cancer	6 of 6 APs hyperplasia or PIN, 6 of 6 APs invasive cancer

a Ad-CMV-Luc recipient mice.

**FIGURE 4:**
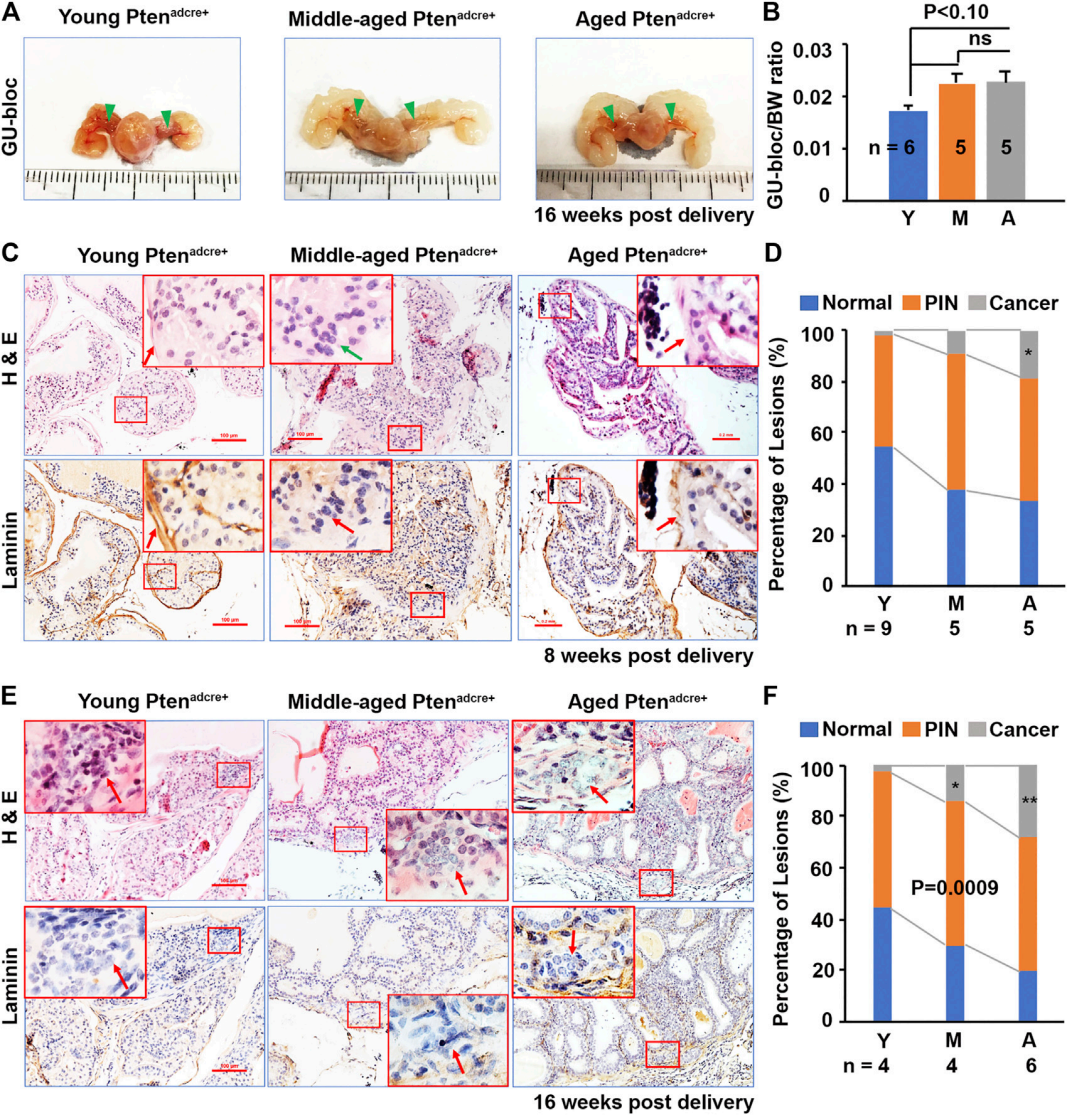
Aged *Pten* ^*adcre+*^ mice have increased GU-bloc/body weight ratio and PCa onset and progression compared to young mice. **(A)** Representative photographs of the GU-blocs; GU‐bloc, genitourinary bloc; arrowheads indicate the APs. **(B)** GU-bloc/body weight (BW) ratio; ns, non-significant. **(C)** Representative serial sections of anterior prostatic lobes stained with H&E and or for laminin 8 weeks p.d.; arrows indicate the invasive site in middle-aged mice (loss of laminin staining) and non-invasive sites in young (obvious laminin staining) and aged mice (reduced laminin staining). **(D)** Percentages of normal, PIN and cancer (invasive prostate adenocarcinoma) in anterior prostatic lobes of young, middle-aged, and aged mice 8 weeks p.d.; *p < 0.05. **(E)** Representative serial sections of APs stained with H&E and or for laminin 16 weeks p.d.; arrows indicate the invasive sites in the three groups of mice. **(F)** Percentages of normal, PIN and invasive cancer in APs of young, middle-aged, and aged mice 16 weeks p.d.; *p < 0.05.

At 8 weeks p.d. groups, the premalignant phenotype became much more evident and the prostatic epithelia displayed increased cell size, nuclear atypia, and abnormal cellular morphology (Ratnacaram et al., 2008; Figure 3Aiii; Figure 4C). In the young group, 16 of 18 APs displayed hyperplasia and PINs, 2 of 18 APs displayed microinvasive cancer (Table 1); by counting the prostate glands (see *Materials and Methods*), ∼41% of prostate glands displayed PINs plus microinvasive cancer (Figure 4C, left column). In the middle-aged group, 7 of 8 APs displayed hyperplasia or PIN, 4 of 8 APs displayed microinvasive cancer (Table 1); ∼49% of prostate glands displayed PINs plus microinvasive cancer (Figure 4C, middle column). In the aged group, all the mice APs developed hyperplasia and PIN and invasive cancer (Table 1) and ∼73% of glands displayed PINs plus micro-invasive cancer (Figure 4C, right column).

At 16 weeks p.d. groups, PIN lesions were developed in almost all of the glands. Some PIN lesions had progressed to microinvasive and invasive adenocarcinomas (Figures 3Aiv,Bvii,viii; Figure 4E). In the young group, three of 4 APs displayed microinvasive cancer (Table 1); ∼64% of glands displayed PINs plus microinvasive cancer (Figure 4E, left column). In the middle-aged group, 3 of 4 APs displayed invasive cancer (Table 1); ∼81% of glands displayed PINs plus micro or invasive cancer (Figure 4E, middle column). In the aged group, all the APs displayed invasive cancer (Table 1); ∼93% of glands displayed PINs plus invasive cancer (Figure 4E, right column).

### Aged and Middle-Aged *Pten* ^*adcre+*^ Mice Developed Larger GU-Bloc and GU-Bloc/Body Weight Ratio and Increased Invasive Cancer Than Young *Pten* ^*adcre+*^ Mice

To determine the effect of Pten ablation in mouse PCa development at different ages, we compared the GU-bloc and body weight between aged and non-aged mice. The GU‐bloc weight is proportional to the prostate weight and is often been used to represent prostate tumor burden (Liu et al., 2020). Representative GU‐blocs of young, middle‐aged, and aged *Pten* ^*adcre+*^ mice are presented in Figure 4A, showing that the GU‐blocs of middle‐aged and aged *Pten* ^*adcre+*^ mice were clearly larger than those of young *Pten* ^*adcre+*^ mice. Compared to young mice, the middle‐aged and aged *Pten* ^*adcre+*^ mice also had significantly increased body weight (BW) and GU‐bloc weight (data not shown). After normalizing the GU‐bloc weight to the corresponding body weight, the middle-aged and aged *Pten* ^*adcre+*^ mice showed a marginally significant increased GU‐bloc/BW ratio compared to the young *Pten* ^*adcre+*^ mice (*p* < 0.10), while there was no significant difference between middle-aged and aged *Pten* ^*adcre+*^ mice (Figure 4B). Compared to the young *Pten* ^*adcre+*^ mice, which had normal prostatic epithelia, the middle‐aged and aged *Pten* ^*adcre+*^ mice had increased prostatic hyperplasia, PINs, and invasive cancer at 8 weeks p.d. groups. Figure 4C shows the representative alterations in the APs of 8 weeks p.d. groups, young mice showed hyperplasia lesion with complete laminin staining; aged mice exhibited PIN lesions but with reduced laminin, and middle-aged mice exhibited invasive lesions with loss of laminin staining (arrow). At the 16 weeks p.d., both H&E-stained sections and laminin-stained sections showed invasive lesions (arrows). Quantitative evaluation revealed differences in the incidence of invasive adenocarcinoma between aged (11.54%) and young mice (3.39%), and between middle-aged (10.93%) and young mice at 8 weeks p.d. groups (*p* = 0.0092 and *p* = 0.042 respectively), between aged (34.04%) and young (2.75%), and between middle-aged (14.56%) and young at 16 weeks p.d. groups (*p* = 0.0022 and *p* = 0.023 respectively).

### Increased Cellular Proliferation and Decreased Apoptosis in the Epithelium of Aged *Pten* ^*adcre+*^ Mice Compared to Young Mice Are Related to More Activated P-S6 Positive Cells

**FIGURE 5:**
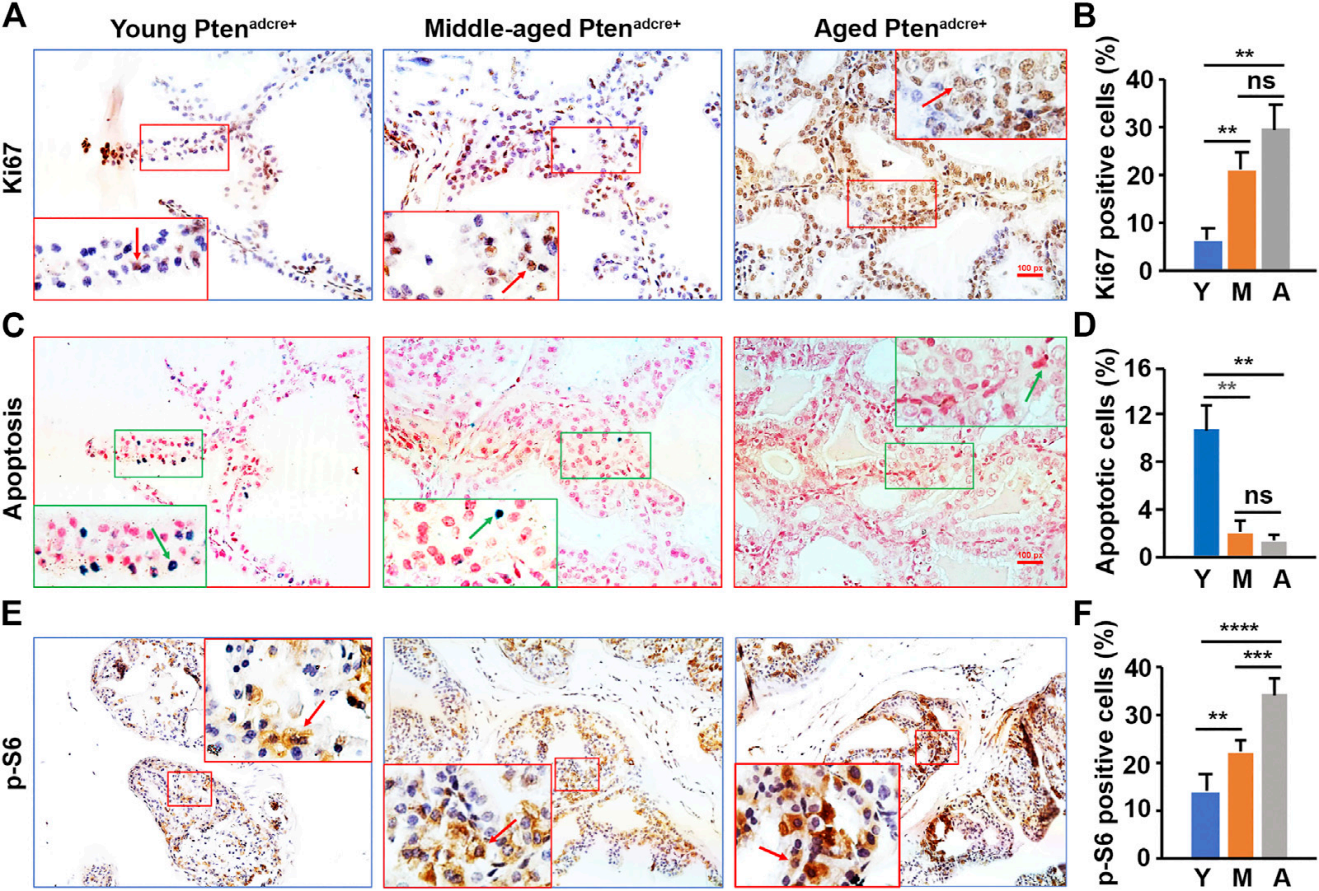
Aged *Pten* ^*adcre+*^ mice have significantly increased activated P-S6, increased cellular proliferation, and reduced apoptosis in prostatic epithelial cells compared to young mice. **(A)** Representative prostate sections stained for Ki-67 at 16 weeks p.d. at different ages; arrows indicate the positive cells. **(B)** Percentages of Ki-67-positive cells in the mouse APs; data are represented as mean ± SEM; *n* = 3 animals per group; **p < 0.01; ns, non-significant. **(C)** Representative prostate sections stained for apoptotic cells at 16 weeks p.d. at different ages; arrows indicate the positive cells. **(D)** Percentages of apoptotic cells in the mouse APs; data are represented as mean ± SEM; *n* = 3 animals per group; **p < 0.01; ns, non-significant. **(E)** Representative IHC staining of the P-S6 protein in the AP tissues of *Pten* ^*adcre+*^ mice 16 weeks p.d. at different ages; arrows indicate positive cells. **(F)** Percentages of P-S6-positive cells in the mouse APs; data are represented as mean ± SEM; *n* = 3 animals per group; **p < 0.01; ***p < 0.001; ****p < 0.0001.

To understand why aged mice developed larger prostate tumors than young mice, we assessed cellular proliferation and apoptosis by conducting Ki-67 staining and TUNEL assays (Zhang et al., 2012; Zhang et al., 2014) on the *Pten* ^*adcre+*^ mouse APs. We found significantly more Ki-67-positive epithelial cells in aged and middle-aged *Pten* ^*adcre+*^ mice than young mice (Figures 5A,B). Also, there were significantly fewer apoptotic cells in aged mice APs than in young mice APs (Figures 5C,D). We analyzed the P-S6 positive cells in the *Pten* ^*adcre+*^ mice APs and found significantly increased activated cells in middle-aged and aged mice APs compared to those of young mice (Figures 5E,F). Thus, the increased cellular proliferation and decreased apoptosis in aged mice may be related to the increased activation of P-S6. We also found significantly increased P-AKT and P-4E-BP1 activation in aged and middle-aged mice compared to young mice (data not shown).

### Increased Inflammatory Cell Infiltration in the Stroma of Aged *Pten* ^*adcre+*^ Mice Compared to Young Mice

**FIGURE 6:**
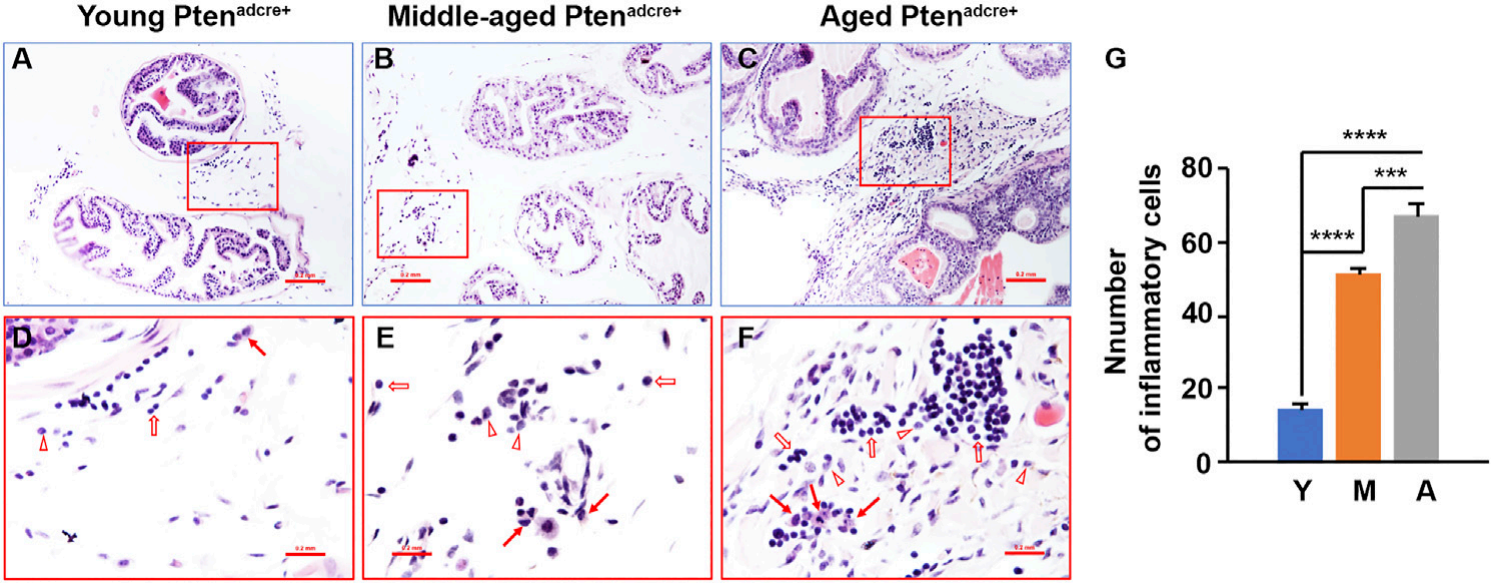
Aged *Pten* ^*adcre+*^ mice have significantly increased inflammatory cell infiltration compared to young mice. **(A–C)** Representative sections of anterior prostatic lobes stained with hematoxylin and eosin at different ages; original magnification, ×100. **(D–F)** ×400 magnification of the selected regions in **(A–C)**; arrows indicate macrophages, open arrows indicate lymphocytes, and arrowheads indicate neutrophils. **(G)** Comparison of the numbers of inflammatory cells per high‐power field; ***p < 0.001; ****p < 0.0001; Y, Young Pten^adcre+^; M, Middle-aged Pten^adcre+^; A, Aged Pten^adcre+^.

To understand the molecular mechanisms underlying aged *Pten* ^*adcre+*^ mice which had increased onset and progression of prostate adenocarcinoma, we examined the inflammatory cell infiltration in the prostate stroma. We found that middle-aged and aged mice had many inflammatory cells in the connective tissue space between the prostatic glands compared to young mice (Figures 6A–G). The inflammatory cell populations were mainly lymphocytes and macrophages, with few neutrophils (Figures 6D–F).

## Discussion

There are fundamental differences between human and mouse prostate biology and tumorigenesis (Grabowska et al., 2014). Human males have a lifetime risk of one in six for developing PCa, but mice rarely develop spontaneous PCa (Suwa et al., 2002). In addition, there are other key differences between human and mouse prostate anatomy and physiology that are not represented in current PCa models. GEMMs have proven to be remarkably predictive for human PCa (Arriaga and Abate-Shen, 2019). Genomic alterations that occur at certain disease stages tend to be modeled in mice. GEMMs have greatly improved our understanding of the pathophysiology of PCa and have provided a valuable platform for testing potential therapeutic strategies. However, to date, no current GEMMs for PCa reflect the impact of age on PCa initiation and progression has been available. Since age is the most important risk factor for PCa, it is necessary to develop age-depenent PCa animal models to better understand human PCa initiation and progression.

Several transgenic mouse lines that express Cre recombinase selectively in the prostatic epithelium to conditionally target floxed genes in the prostate (Maddison et al., 2000; Wu et al., 2001; Jin et al., 2003; Ma et al., 2005) have been valuable for assessing prostate carcinogenesis. However, the major drawback of these models is lack of temporal control of Cre recombinase expression/activity. Ablation of target genes occurs before completion of puberty, before the prostate becomes fully differentiated. As human PCa rarely occurs before 40 years of age, the present murine Cre recombinase models do not accurately recapitulate human PCa. To more closely mimic human PCa initiation and progression, initiation of Cre recombinase expression would be ideal in young adult mice at 4–6 months of age according to mouse to human age scaling using published criteria (Carnes et al., 2003). In addition to the time considerations for generating knock-out GEMMs for PCa studies, aging studies require an extended time to age the mice. The present study developed a novel virus-assisted Pten cKO mouse model, *Pten* ^*adcre+*^, that permits comparison of tumor formation in the same time interval post-Pten deletion between aged and non-aged mice. In this present mouse model, the prostate-specific Cre-LoxP gene switching is generated *via* intraductal delivery of an adenovirus expressing Cre with luciferin tagging (Adeno-Cre-Luc) to the APs. Ad-CMV-Luc is injected as a control. This approach saves time and allows investigators to confirm the successful delivery in the mouse prostatic duct by live imaging post-delivery.

The present model, in which virus-assisted Cre-expression-mediated biallelic ablation of the Pten gene at different ages can initiate prostate carcinogenesis, closely mimics the course of human PCa formation. The focal hyperplasia of epithelial cells occurs at 4 weeks post-Pten ablation. By 8 weeks, the PIN lesions have grown in the prostate epithelium. Some PINs then progress to microinvasive adenocarcinoma at 8 weeks post-Pten ablation. The invasive adenocarcinoma occurs and increases at 16 weeks post-Pten ablation. Importantly, the prostate carcinogenesis initiated in middle-aged and aged mice develops with significantly more rapid onset and progression of PCa compared to that initiated in young mice. The underlying mechanisms are related to increased cellular proliferation, decreased apoptosis, and increased inflammatory cell infiltration in the prostatic stroma.

The current model exhibits similarities to previously established mouse models in which Pten was selectively ablated in the prostatic epithelium by using transgenic mice expressing the Cre recombinase under the control of a composite probasin promoter (PB-Cre4) (Trotman et al., 2003; Wang et al., 2003) or the PSA promoter (PSA-Cre) (Ma et al., 2005). Although the kinetics and extent of tumor progression were different in these models, Pten ablation drives the initiation and progression of prostate adenocarcinoma *via* the development of hyperplasia, PINs, to invasive cancer. Compared to these GEMMs, the tamoxifen-inducible Cre lines showed a slowed pace of disease progression (Luchman et al., 2008a; Ratnacaram et al., 2008). The present model are consistent with these inducible lines, which exhibit a similar hyperplastic development pace at about 4 weeks post virus delivery. However, by 8 weeks p.d. of the virus, PINs developed and microinvasive cancer appears. The pace is quicker than the tamoxifen-inducible lines. Pten ablation in the present model is also related to the time of post virus delivery. Interestingly, we compared different doses of Cre-expressing virus injection and found that the Pten ablation is dose-dependent (data not shown). We used the same dosage of Cre recombinase delivered to the different aged mice as our goal in this study was to emphasize the effect of aging on prostate carcinogenesis. In this way, we were able to compare the infection efficiency, Pten ablation, downstream signaling activation, and onset and progression of prostate adenocarcinoma at different ages.

The present model demonstrated a direct correlation between Pten deficiency and P-AKT/PS6K1/P-4E-BP1 activation with development of pre-neoplastic epithelial lesions. This is consistent with a previous tamoxifen-inducible line (Luchman et al., 2008a) that showed a direct correlation between Pten deficiency and P-S6 ribosomal protein upregulation in preneoplastic cell lesions. However, our study goes well beyond this as we generated Pten ablation in mice at different ages (aged vs. non-aged). Most significantly, we found that middle-aged and aged mice with Pten ablation had significantly increased activation of the PI3K/AKT/mTOR/S6K and PI3K/AKT/mTOR/4EBP1 axes compared to younger mice. These results suggest that aged mice exhibited similar Pten mutation efficiency with a similar virus infection but leads to significant activation of the PI3K/AKT/mTOR pathways in the prostate epithelium.

In summary, we have generated a novel virus-assisted spatially and temporally controlled PCa mouse model to address the role of aging in prostate carcinogenesis. Using this model, we can compare tumor growth in the same time interval post-Pten excision between aged and non-aged mice. Furthermore, the role of specific genes in Pten-related carcinogenesis during aging can be evaluated by first crossing knock-out mice for the specific gene with the Pten^L/L^ mice, and then, using adenoviral vectors, precisely deliver Cre-recombinase to specific tissues in a temporally manageable manner (Leow et al., 2005). In addition, the present age-dependent carcinogenesis model can be used to test efficacy of pharmacological agents in the prevention/treatment of cancer in aged vs. non-aged mice.

## Conclusion

The prostate-specific *Pten* KO (*Pten* ^*adcre+*^) mouse model can be induced at different ages by the adenovirus-assisted *in vivo* conditional KO approach. This model allows comparison of tumor growth in the same time interval post-*Pten* excision between the aged and non-aged mice, leading to a better understanding of the effects of aging on prostate carcinogenesis.

## Data Availability

The original contributions presented in the study are included in the article/Supplementary Material, further inquiries can be directed to the corresponding author.

## Supplementary Material

**SUPPLEMENTARY FIGURE 1** | The PI3K signaling pathway downstream component is activated in the APs of Pten^L/L^ mice post Cre-expressing adenovirus delivery. **(A-B)** Serial sections show that Pten loss in the prostatic epithelium is associated with an increase in P-AKT protein levels in the preneoplastic phase in *Pten^adcre+^* mice; arrows indicate cells that lost Pten and had activated P-AKT cells.

**SUPPLEMENTARY FIGURE 2** | The efficiency of adenovirus-assisted Cre-mediated excision of floxed Pten gene. The relative amount of Pten mRNA expression was determined by qRT-PCR on mRNA isolated from 16 weeks post Ad-Cre-Luc and Ad-CMV-Luc delivered mouse prostate anterior lobes. Ad-Cre-Luc, mice 16 weeks post-delivered Ad-Cre-Luc; Ad-CMV-Luc, mice 16 weeks post-delivered Ad-CMV-Luc.
