# Supplementary figures and images for: A Novel Controlled PTEN-Knockout Mouse Model for Prostate Cancer Study
Source: Front Mol Biosci. 2021 Jun 3;8:696537. doi: 10.3389/fmolb.2021.696537 (PMC8211560; doi:10.3389/fmolb.2021.696537)

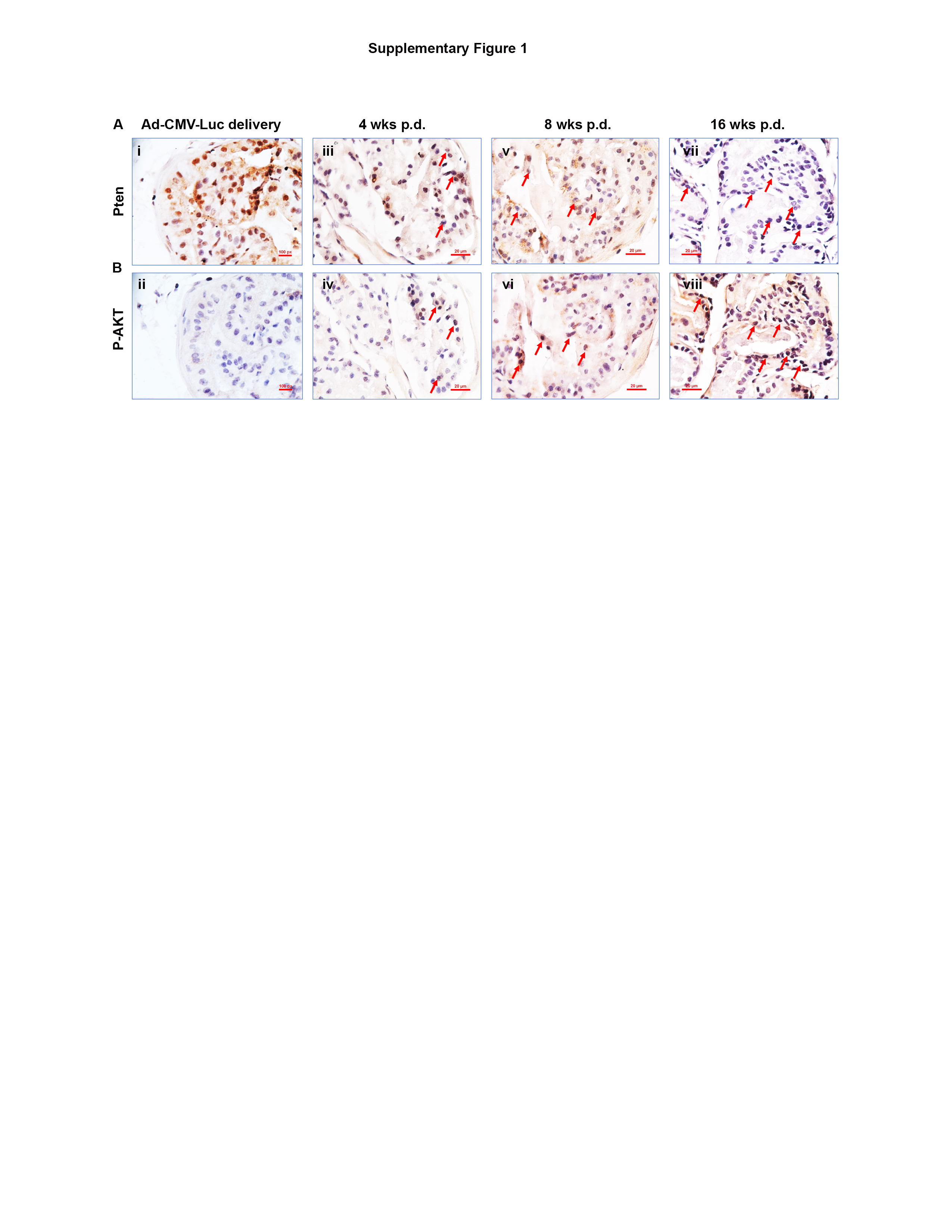

Supplement: Supplementary file 1 [file image1.jpeg]

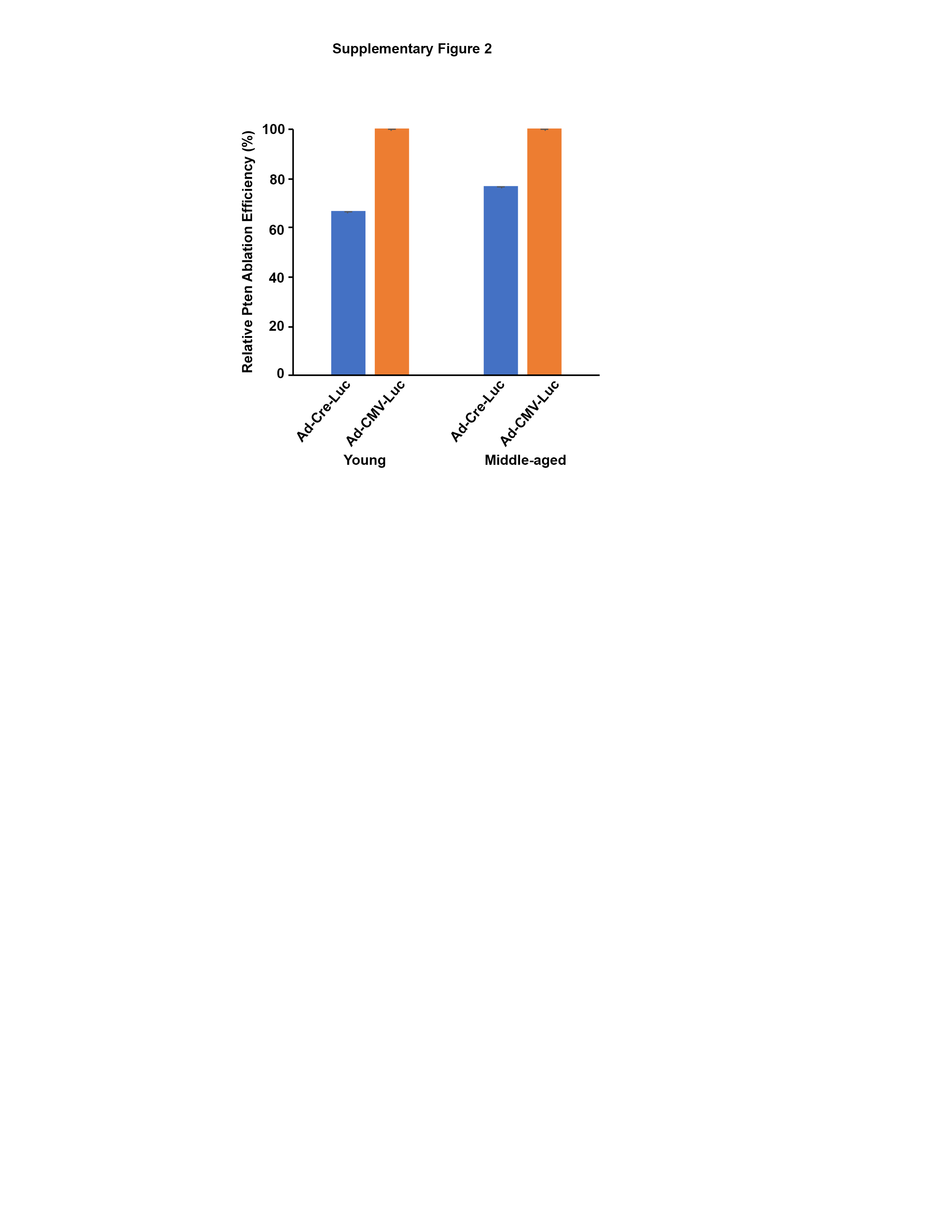

Supplement: Supplementary file 2 [file image2.jpeg]
